# *Portulaca oleracea* L. Methanolic Extract Protects the Brain of Male Rats Against Alzheimer's Disease

**DOI:** 10.1155/sci5/7701263

**Published:** 2025-02-22

**Authors:** Haddad A. El Rabey, Samar M. Rezk, Seham A. Mahmoud, Zienab Abdallah, Mennatallah Loutfy, Adel I. Alalawy, Yasmene F. Alenazi, Heba Sheta, Nadia Bakry

**Affiliations:** ^1^Biochemistry Department, Faculty of Science, University of Tabuk, Tabuk, Saudi Arabia; ^2^Bioinformatics Department, Genetic Engineering and Biotechnology Research Institute, University of Sadat City, Sadat City, Egypt; ^3^Clinical Nutrition Department, Mahalla Hepatology Teaching Hospital, El-Mahalla El-Kubra, Gharbia, Egypt; ^4^Chemistry Department, Faculty of Science, Zagazig University, Zagazig, Egypt; ^5^Physiology Department, Faculty of Medicine, Mansoura University, Mansoura, Egypt; ^6^Bone Marrow Transplantation and Cord Blood Bank Unit, Children Hospital, Mansoura University, Mansoura, Egypt; ^7^Pathology Department, Faculty of Medicine, Mansoura University, Mansoura, Egypt

**Keywords:** AChE, Alzheimer, IL-6, oxidative stress, *Portulaca oleracea*, TNF-α

## Abstract

Alzheimer's disease (AD) occurs as a result of a chronic neurodegenerative disorder that is most frequently linked to a decline in cognitive function and memory. Twenty-four male rats were divided into four groups (*n* = 6); Group I was the negative control, Group II was the AlCl_3_-positive control, and Group III and Group IV were treated with 100 mg and 200 mg/kg of *Portulaca oleracea* methanolic extract, respectively. Aluminum chloride intoxication in Group II increased lipid peroxidation and decreased antioxidant parameters and affected interleukin-6 (IL-6), the tumor necrosis factor-alpha (TNF-α), acetylcholinesterase (AChE), and amyloid beta (Aβ), which lead to the induction of AD through injuring brain cells of AD rats. Treating the AD rats in Group III (GIII) and Group IV (GIV) with *P. oleracea* ameliorated the altered parameters in the AD rats. It also increased folic acid and vitamin B12 levels. *P. oleracea* modulated the physiological, biochemical, and histological changes brought on by AlCl_3_ intoxication in rats via oxidative stress and inflammatory pathways. The dose of *P. oleracea* in GIV successfully modified the behavioral changes brought on by AlCl_3_ in the AD rats more than that of GIII.

## 1. Introduction

Alzheimer's disease (AD), the most prevalent form of dementia and responsible for about two-thirds of cases of dementia, is typically a neurodegenerative disease of old age with the subtle onset and progressive impairment of cognitive functions, behavior, memory, attention, reasoning, comprehension, language, and judgment [1]. AD cannot be cured, but some treatments can lessen some of the signs [2, 3]. Short-term memory loss was the first disease symptom of AD, which was followed by other progressive disease symptoms such as changes in mood, behavior, aggression, confusion, avoidance of people and social ties, and long-term memory loss [4, 5]. The formation of intracellular neurofibrillary knots and the buildup of amyloid beta (Aβ) peptides in neuronal cells are considered the main histopathological features of AD. Acetylcholinesterase (AChE), which is crucial for proper memory and cognition, is suppressed by A overexpression [6]. Amyloid genesis, oxidative stress, and neuroinflammation were considered to be important indicators for the pathological development of AD [7, 8].

According to many research studies, heavy metals are closely linked to neurodegenerative diseases such as AD [9]. The third-most common and extensively used heavy metal in the world, aluminum (Al), has played a role in the development of neurodegenerative diseases. Because aluminum chloride (AlCl_3_) is present in many professionally produced items, including toothpaste, foods, medicines, and packaged drinking water, its use is extremely complicated [10, 11].

The progression of AD could be slowed down or stopped by taking natural supplements with strong anti-inflammatory and antioxidant qualities [12]. The World Health Organization lists *Portulaca oleracea* L., also known as Rigla (Egypt), as a warm-climate, herbaceous succulent annual shrub [13]. Modern pharmacological research on *P. oleracea* has shown that it is loaded with minerals, omega-3 fatty acids, vitamins, phenolic alkaloids, glutathione, and many other nutrients [14]. In addition, the methanolic extract of *P. oleracea* contains flavonoids, alkaloids (oleracein A, B, and C), phenolic acids, caffeic acid, *p*-coumaric acid, ferulic acid omega-3 fatty acids, α-linolenic acid, saponins, and polysaccharides as analyzed by gas chromatography-mass spectrometry (GC-MS) [15]. So, *P. oleracea* has a broad range of pharmacological characteristics, including those that reduce oxidative stress, have cytotoxic effects on cancer, have antibacterial effects, have neuroprotective effects, and can modulate inflammation [16].

The goal of the current study was to evaluate the potential benefits and pharmacological effects of the methanolic extract of *P. oleracea* against the neurodegenerative features of AD induced by AlCl_3_ intoxication in the rat model.

## 2. Materials and Methods

### 2.1. Chemicals and Reagents

Biochemical investigations of IL-6 (R&D Systems), TNF-α (Abcam), Aβ and AChE (MyBioSource), folic acid, and vitamin B12 (ELISA kit from Cusabio) were carried out according to the manufacturer's instructions. AlCl3, ethanol, and other chemicals were purchased from Sigma-Aldrich Company, USA.

### 2.2. Plant Material Collection and Methanolic Extract Preparation

*P. oleracea* L. was harvested from a nearby field in Mansoura City, Egypt. The plant specimen of *P. oleracea* used in this study was first identified by the plant taxonomist Dr Yasser El-Amier, Faculty of Science, Mansoura University, and a herbarium specimen with No. Mans.0161615004 was deposited in the Herbarium of the Faculty of Science, Mansoura University, Egypt. The plants were cleaned with distilled water and dried, and a plant processor crushed the fresh vegetal material to produce a powder that was then kept at room temperature. The plant's oil was extracted by macerating 500 g of *P. oleracea* in 1500 cc of methanol and shaking for 24 h at room temperature. The extract was concentrated using a rotary evaporator, centrifuged, filtered, and dried at 60°C under pressure. The final sample had a 2.5% (w/w) compared to the original weight of plant material [17].

### 2.3. Experimental Animals and Drug Delivery

Twenty-four adult male Sprague Dawley rats weighing 180 ± 5 g were bought from the Agricultural Research Center in Giza, Egypt. The rats were kept under observation for one week before starting the experiment in stainless steel cages (*n* = 6) in a tidy, well-organized chamber with temperature regulation of 22°C–24°C and a 12-h dark/light cycle. The AlCl_3_ stock solution was prepared by dissolving AlCl_3_ in distilled water (20 mg/mL) and the pH was adjusted to 7.4 with 0.1 M phosphate buffer saline (PBS). The resultant solution is the applied dose of AlCl_3_, which is equivalent to 100 mg/kg.

The rats were divided into four groups as follows: Group I (GI) was the untreated negative control group, Group II (GII) was the positive control group that received 17 mg of AlCl_3_ suspended in 1 mL distilled water as a vehicle intraperitoneally injected once a day for 15 consecutive days to induce AD [18], Group III (GIII) received AlCl_3_ as in GII and then treated daily with *P. oleracea* methanolic extract orally by stomach gavage at a dose of 100 mg/kg per day for 45 days, and Group IV (GIV) received AlCl_3_ as in GII and then treated daily with 200 mg/kg of *P. oleracea* methanolic extract orally by stomach gavage for 45 days. The experimental work was conducted under the approved policy and guidelines of the Ethics Committee of the Faculty of Medicine at Mansoura University, Egypt, Key Number: MU-ACUC (SC.R.23.03.11), for 60 days. All rats were fed a regular basal rodent diet. Feed and water were provided *ad libitum* during the experiment period.

### 2.4. T-Maze Test for the Estimation of Cognitive Capabilities

According to Deacon and Rawlins' method [19], the T-maze test was used to estimate the rat's cognitive capacity. The animals were given only water to drink for 24 h before the experiment. The T-maze test was administered to the four groups at the following times: (a) time zero, before oral induction with AlCl_3_, (b) after the AD induction phase had passed by 24 h, and (c) 24 h after the final oral treatment with the test substances. Before and after the experiment, behavioral observations were made and documented.

### 2.5. Blood Sampling and Tissue Collection

At the end of the experimental period, the rats were weighed and euthanized according to the protocol of AVMA (2020) in their cages using carbon dioxide without removing them from their home cages to prevent stress caused by handling. The CO_2_ flow was applied to displace 30%–70% of the cage volume per minute, which caused narcosis to the animals. This was followed by cervical dislocation and then dissection. Each animal's entire brain was quickly removed, cleaned with isotonic saline, dried, weighed, and homogenized for additional calculations [20]. To acquire sera and plasma, blood samples were drawn and centrifuged for 10 min at 3000 rpm at 4°C. They were then stored until assays could be run.

### 2.6. Determination of Biochemical Parameters

Serum levels of IL-6 (R&D Systems rat kit), TNF-α (Abcam rat kit), Aβ and AChE (MyBioSource rat kit), folic acid (Cusabio rat kit), and vitamin B12 (Lifespan BioScience rat kit) were examined according to the manufacturers' instruction.

### 2.7. Antioxidant Markers and Oxidative Stress Estimation

Serum catalase (CAT), superoxide dismutase (SOD), total antioxidant capacity (TAC), and malondialdehyde (MDA) were measured according to the methods described by Chance and Mackley [21], Dechatelet et al. [22], Koracevic et al. [23], and Stocks and Dormandy [24], respectively.

### 2.8. Histopathological Study

Following their overnight fixation in 10% formalin, brain specimens from the cerebral cortex including the hippocampus portions were histologically processed to obtain paraffin sections (4–6 mm thick). The sections were stained with hematoxylin and eosin and lastly photographed beneath the light microscope attached to a camera.

### 2.9. Statistical Analysis

Data were analyzed using SPSS software (version 16.0), and the findings are presented as mean ± standard deviation. One-way ANOVA was used to analyze statistical differences between groups. The Pearson correlation coefficient test and statistical significance were defined as *p* > 0.05.

## 3. Results

### 3.1. AChE and Aβ Contents in Brain Tissues of AlCl_3_-Induced AD Rats

**Figure 1:**
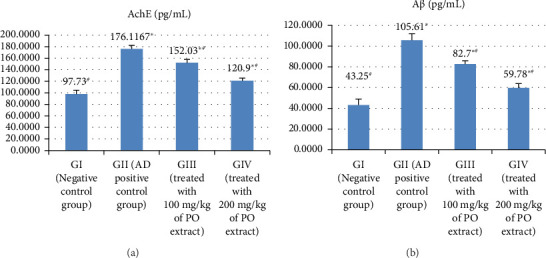
The effect of Alzheimer's induction and treatment with the methanolic extract on AchE (a) and Aβ (b) in the studied rat groups.

Figures 1(a) and 1(b) and Supporting Table S1 show that AlCl_3_-induced AD rats in GII had substantially higher AChE and Aβ levels in their brains than control rats of GI (at *p* > 0.05). The AChE and Aβ of AD animals in GIII and GIV were noticeably suppressed by the methanolic extract of *P. oleracea* therapy. In addition, it was found that the methanolic extract of *P. oleracea* 200 mg/kg dose-treated group (GIV) had significantly lower AChE and Aβ levels than the methanolic extract of *P. oleracea* 100 mg/kg dose-treated group (GIII) (at *p* > 0.05).

### 3.2. The Inflammatory Markers (TNF-α and IL-6)

**Figure 2:**
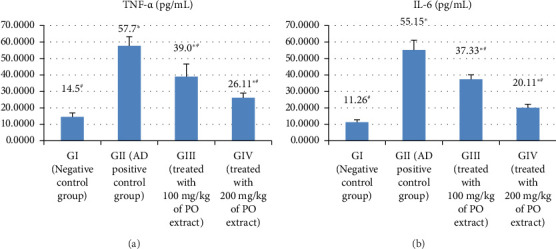
The effect of Alzheimer's induction and treatment with the methanolic extract on TNF-α (a) and IL-6 (b) in the studied rat groups.

TNF-α and IL-6 levels, which are depicted in Figures 2(a) and 2(b) and Supporting Table S1, were significantly higher (at *p* > 0.05) compared to the negative control group (GI). The current findings were intriguing in that they demonstrated that various concentrations of the methanolic extract of *P. oleracea* in GIII and GIV could significantly reduce the expression of these markers when administered to cells (*p* > 0.05). In addition, when compared to the low dose (100 mg/kg) of the methanolic extract of *P. oleracea*-treated group, 200 mg/kg of the methanolic extract of *P. oleracea* in GIV significantly decreased both TNF-α and IL-6 expressions, although it was still significantly higher than the control (*p* > 0.05).

### 3.3. Serum Folic Acid and Vitamin B12 Concentration

**Figure 3:**
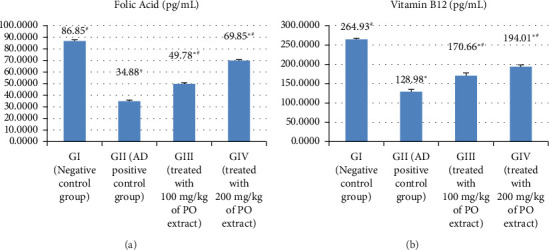
The effect of Alzheimer's induction and treatment with the methanolic extract on folic acid (a) and vitamin B12 (b) in the studied rat groups.

Folic acid and vitamin B12 concentrations were considerably lower in the AlCl_3_-treated rats in GII than in the negative control group (GI) (at *p* > 0.05), as shown in Figures 3(a) and 3(b) and Supporting Table S1. When compared to AlCl_3_-induced AD rats in GII, groups receiving a methanolic extract of *P. oleracea* in GIII and GVI exhibited a significant increase in folic acid and vitamin B12 (at *p* > 0.05).

### 3.4. Oxidative Stress and Antioxidant Markers

**Figure 4:**
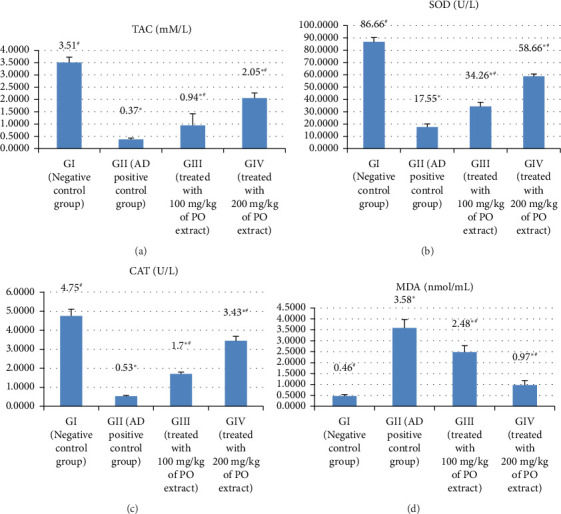
The effect of Alzheimer's induction and treatment with the methanolic extract on TAC (a), SOD (b), CAT (c), and MDA (d) in the studied rat groups.

A significant decline in the activities of the antioxidant enzyme catalase, SOD, and TAC was observed in AlCl_3_-induced AD group (GII) (*p* > 0.05), as shown in Figures 4(a), 4(b), and 4(c) and Supporting Table S2. However, compared to GII, TAC, SOD, and CAT activities were significantly higher in the methanolic extract of *P. oleracea*-treated groups (GIII and GIV) (at *p* > 0.05). Additionally, a significant increase was observed in these biomarkers treated with 200 mg/kg methanolic extract of *P. oleracea* in GIV as compared to low-dosage methanolic extract of *P. oleracea* in GIII (*p* > 0.05) (Table 1).

In addition, significant variations between the research groups were discovered to be present in Figure 4(d) and Supporting Table S2. When compared to the normal rats, lipid peroxidation (MDA) was considerably higher in the AD-induced group (GII). The methanolic extract of *P. oleracea*-treated groups displayed a significant decrease in MDA elevation (at *p* > 0.05). When compared to the low-dosage methanolic extract of the *P. oleracea-*treated group (GIII) (100 mg/kg), MDA significantly decreased in the high-dose methanolic extract of *P. oleracea*-treated group (GIV) (200 mg/kg) (at *p* > 0.05).

### 3.5. Effect on T-Maze Activity (Behavior Stress Maze)

When compared to the negative control group (GI), AlCl_3_-induced AD group rats in GII took significantly longer (by seconds) to reach the food in the T-maze test, indicating a breakdown in neurocognitive function (*p* > 0.05). Additionally, compared to the AD-induced group (GII), the methanolic extract of *P. oleracea*-treated groups (GIII and GIV) took significantly less time to arrive at the assignment (at *p* > 0.05), demonstrating improved cognitive abilities (Table 2).

### 3.6. Correlation Among the Studied Biomarkers

Positive correlations were found between the studied biomarkers CAT, SOD, folic acid, and vitamin B12 in addition to a potential link between the various biomarkers. On the other hand, measurements of antioxidants, folic acid, and vitamin B12 revealed adverse and significant associations between TNF-α and IL-6 and these substances. TNF-α, IL-6, Aβ, and AChE were observed to have a positive and highly significant correlation, according to the observed data from this research (Table 1).

### 3.7. Effect of *P. oleracea* on the Brain Histopathology of the AlCl_3_-Induced AD Groups

**Figure 5:**
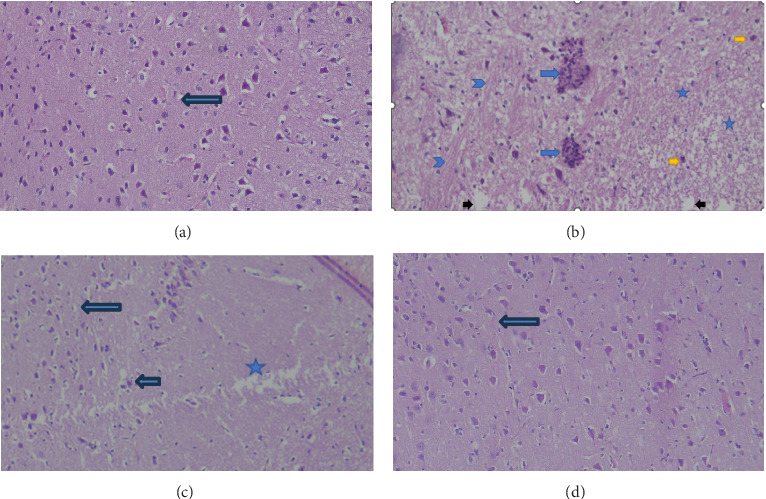
The effect of *Portulaca oleracea* L. on the brain histopathology of the AlCl_3_-activated AD rats. (a) Control rats exhibited the typical histological structures of the brain (arrow) (GI). (b) The AlCl_3_-triggered AD rats demonstrated neuronal degeneration (yellow arrows) with absence of neurons (stars), edema (black arrows), microglial nodules (blue arrows) and glial fibers (arrowhead) (GII). (c) Rats were given AlCl_3_ as in GII and concurrently treated with *P. oleracea* methanolic extract at a dose of 100 mg/kg per day for 15 days that show remnant of brain tissue with degenerative changes and absence of neurons (star) and near-normal brain tissue with mild reactive gliosis (long arrow) and inflammatory cellular infiltrate (short arrow) (GIII). (d) Rats were given AlCl_3_ as in GII concurrently treated with 200 mg/kg of *P. oleracea* methanolic extract daily for 15 days near-normal brain tissue with no areas of neuronal loss (X100). Hematoxylin and eosin (X200, H&E).

Figure 5 shows the results of the histopathology examination of brain sections from all groups. The brains of normal control animals (Figure 5(a)) showed normal neurons, fibers, and hippocampus portions. Conversely, the brains of the control positive animals injected with AlCl_3_ (Figure 5(b)) showed marked neuronal degeneration associated with the absence of neurons (stars), edema, microglial nodules, and glial fibers. On the other hand, the brains of diseased animals treated with low-dosage *P. oleracea* showed degenerative changes and absence of neurons and near-normal brain tissue with mild reactive gliosis and inflammatory cellular infiltration (Figure 5(c)). Moreover, the brains of diseased animals treated with high-dosage *P. oleracea* showed near-normal brain tissue with no areas of neuronal loss (Figure 5(d)). The *P. oleracea*-treated animals also demonstrated protective actions as evidenced by the reduced histological alterations and typical normal cellular structures.

## 4. Discussion

AD is a heterogeneous disease with complex pathobiology and is a growing medical catastrophe that has a significant emotional and financial toll on those who have it and their families [25]. The cognitive impairments associated with AD have a variety of strong etiologies that include the characteristics of Aβ plaques and neurofibrillary tangles, excessive reactive oxygen species (ROS) generation, and inflammatory cytokine molecules production, such as tumor necrosis factor (TNF-α) and interleukin (IL)-6 that has a broad range of pharmacological properties, including neuroprotective, antimicrobial, antidiabetic, antioxidant, and anti-inflammatory [26]. Additionally, acetylcholine (ACh) degradation is increased when Aβ is overexpressed, and the inflammatory molecules IL-6 and TNF-α have been used as defining markers for the neuropathological diagnosis of AD [13, 27]. These findings are consistent with our current research, which shows that AD rodents were induced to produce more A, Ach, IL-6, and TNF-α.

Malnutrition is involved in the pathogenesis of AD, according to mounting evidence [28]. Healthy eating and a mix of nutrients, including vitamins, are therefore essential for preventing AD [29]. In addition, mounting evidence suggests that metal toxicity, such as aluminum, is linked to neurological diseases, with aluminum being the most potent neurotoxin [30]. This supports our findings that administering AlCl_3_ to AD-induced rats caused induced cholinergic impairment. Many studies suggested that natural supplements with antioxidant and anti-inflammatory properties could help control oxidative stress and inflammation and slow or stop the progression of AD [12]. Ancient Egypt cultivated and utilized the well-known edible annual herb *P. oleracea* as a therapeutic plant [31]. A broad range of pharmacological properties, including neuroprotective, antidiabetic, antimicrobial, anti-inflammatory, antioxidant, anticancer, and antiulcerogenic, are present in *P. oleracea* [32].

In the current study, AlCl_3_-induced AD in rats was evaluated for the antioxidant effects of *P. oleracea* methanolic extract by measuring SOD, CAT, and TAC, as well as the inhibition of oxidative stress response by reducing the MDA content. As compared to the control group, we observed a substantial increase in lipid peroxidation (MDA) and a decrease in the activity of SOD, CAT, and TAC in the AD-induced groups. The results also showed that treatment with a high (200 mg/kg) concentration of the methanolic extract of *P. oleracea* was superior to a low dose (100 mg/kg), with MDA levels substantially reduced and CAT, TAC, and SOD significantly increased. These findings are consistent with those of another researcher who reported that the administration of AlCl_3_ raised the MDA level while concurrently reducing the activity of the antioxidant SOD [33–36]. *P. oleracea*'s components, including gallo-polyphenols, omega-3 fatty acids, -tocopherols, ascorbic acid, quercetin, kaempferol, and apigenin, are responsible for the plant's antioxidant properties [37–39]. It also contains polyphenols, such as phenolic acids and flavonoids, which exhibit more powerful antioxidant properties than some naturally occurring antioxidants such as vitamin C and vitamin E [40].

At the cellular level, the brains of rats injected with AlCl_3_ showed marked neuronal degeneration associated with severe vacuolation of neuropil. In agreement, other studies showed neuronal degeneration after injection of AlCl_3_ [6]. In contrast, the brains of diseased animals treated with *P. oleracea* showed mild neuronal degeneration with limited neuronal vacuolation and nuclear pyknosis, indicating neuronal improvement due to the therapeutic activity of the constituents of *P. oleracea* such as alkaloids such as oleracein A, B, and C that are characterized by their neuroprotective and anti-inflammatory activities, phenols that have antioxidant and free radical scavenging activity, and saponins that has anti-inflammatory activity [15]. These ameliorative findings coincided with the previous report mentioned by Wanyina et al. [41].

According to the study's findings, there was a significant increase in AchE activity compared to the control group and a large cholinergic impairment in AD, which can be brought on by Aβ buildup. These findings are in agreement with those of other researchers who found that AlCl_3_ administration caused a substantial increase in AChE activity in the brain, a sign of cholinergic neuron loss in comparison to neurologically healthy control rats [42, 43]. These results concur with earlier findings that ensure high content of phytochemicals including polyphenols, flavonoids, and alkaloids of the methanolic extract of *P. oleracea*, which play an important role as an antioxidant with a possible potential role in the clinical treatment of AD. Using the methanolic extract of *P. oleracea* as a therapy in AD-induced groups showed a decrease in the A content and AChE activity as compared to the untreated AD group [43, 44]. In addition, *P. oleracea*'s alkaloidal extract substantially reduced AChE activity. *P. oleracea* is an efficient agent for AD prophylaxis and treatment because AChE inhibitors have been used as a potential AD treatment [44].

The current study findings demonstrated that AlCl_3_ significantly increased IL-6 and TNF-α levels in AD rodents, while the methanolic extract of *P. oleracea* treatment significantly decreased IL-6 and TNF-α levels. The current study is in line with several others [44, 45] stating that *P. oleracea* L. ethanol extract effectively prevents ulcerative colitis caused by dextran sulfate sodium by inhibiting oxidative stress response through the MDA, NO, and SOD activities, as well as by lowering the mRNA expression of proinflammatory cytokines TNF-α, IL-1, and IL-6. Additionally, this research supports the findings of Bian et al. that kaempferol from *P. oleracea* inhibits NF-B, TLR4, and immediate signaling to block LPS from inducing the expression of the inflammatory mediators TNF-α, IL-6, ICAM-1, IL-1, and VCAM-1.

In addition, the current findings are consistent with other findings, which showed a connection between oxidative stress (MDA), measured inflammatory cytokines (IL-6, TNF-α), and AchE. In the methanolic extract of *P. oleracea*-treated AD groups, we also discovered a substantial downregulation of the proinflammatory cytokines IL-6 and TNF-α. The anti-inflammatory and antioxidant properties of the methanolic extract of *P. oleracea* are supported by these findings. In aging mice, the alkaloid compound betacyanin's soluble pigments had neuroprotective benefits by lowering levels of lipid peroxidation and reactive oxygen species damage [46].

The current study showed that AlCl_3_ induction led to a substantial decline in folic acid and vitamin B12 levels (*p* 0.05), which may have an impact on cognitive behavior by causing systemic inflammation. The current findings show a significant decrease in folic acid and vitamin B12 in rats after AlCl_3_ administration, which may accelerate or advance the progression of AD because it affects cognitive performance. Additionally, the methanolic extract of *P. oleracea* was shown to significantly increase folic acid and vitamin B12 (*p* 0.05) to minimize the development of AD behavior, and this research found agreement with another study that claimed *P. oleracea* is rich in multivitamins, -tocopherol, and B-complex vitamins [47]. This outcome is also consistent with research by Hobbenaghi et al. [48] who found that *P. oleracea* is a great source of vitamin B12, which is crucial for its ability to absorb reactive oxygen species. According to reports, systemic vitamin B12 administration accelerated the healing from peripheral nerve damage in experimental rats.

The current findings also showed that AlCl_3_ greatly reduced the level of folic acid in the AD group. Through systemic inflammation, low or declining folate concentrations may have detrimental impacts on cognitive function. The negative link between TNF-α, IL-6, and folic acid levels must therefore be distinguished. By upregulating antioxidant synthesis, downregulating TNF-α and IL-6 to decrease inflammation, and upregulating folic acid levels, we discovered that the methanolic extract of *P. oleracea* can retract oxidative stress [49]. Therefore, using a large dose of the methanolic extract of *P. oleracea* may improve immune response and advance AD status.

In order to assess both the initial functional symptoms and more stabilized test results, the T-maze was used in this research to measure neurocognitive function at zero time before beginning oral AlCl_3_ induction, followed by 24 h, and finally 24 h after the last oral different doses of the methanolic extract of *P. oleracea*. AlCl_3_ injections demonstrated a substantial decline in attitude estimation at study's beginning, supporting the findings of the authors in [50]. After 15 days, rats treated with 200 mg/kg of EEPO displayed a decline in the discernible effect [51]. Rats could explore the alternate arms of the T-maze, a finding that was also supported by the study of Wu et al. [52], whereas hippocampal-lesioned rats tended to adopt a side preference, resulting in lower spontaneous alternation ratios.

## 5. Conclusion

The powerful growth of AlCl_3_ toxicity in the studied animals disappeared with the treatment of *P. oleracea* methanolic extract. The protective and nutritional activity of *P. oleracea* is ascribed to its rich constituents such as phenols (antioxidant and free radical scavenging activity), alkaloids (oleracein A, B, and C are characterized by their neuroprotective and anti-inflammatory activities), saponins (antimicrobial, anti-inflammatory, and cholesterol-lowering activities), and polysaccharides (immune-boosting and wound-healing properties). These bioactive constituents of *P. oleracea* have the potential to be a genuinely functional food that improves health by reducing aluminum toxicity and be taken into consideration for long-term studies on Alzheimer's prevention and treatment, opening up new possibilities of using *P. oleracea* as phytotherapy in medical approaches. Hopefully, natural therapeutics will improve AD disease monitoring and comprehension soon.

## Figures and Tables

**Table 1:** Correlations coefficient (r) values in some measured parameters in all groups.

	IL-6	TNF-α	Aβ	AchE	Folic acid	Vitamin B12	MDA	TAC	SOD	CAT
IL-6	1	0.941⁣^∗∗^	0.958⁣^∗∗^	0.955⁣^∗∗^	−0.964-⁣^∗∗^	−0.923⁣^∗∗^	0.951⁣^∗∗^	−0.915⁣^∗∗^	−0.946-⁣^∗∗^	−0.961-⁣^∗∗^
TNF-α	0.941⁣^∗∗^	1	0.947⁣^∗∗^	0.921⁣^∗∗^	−0.925⁣^∗∗^	−0.927⁣^∗∗^	0.917⁣^∗∗^	−0.896⁣^∗∗^	−0.934-⁣^∗∗^	−0.932-⁣^∗∗^
Aβ	0.958⁣^∗∗^	0.947⁣^∗∗^	1	0.958⁣^∗∗^	−0.959⁣^∗∗^	−0.941⁣^∗∗^	0.949⁣^∗∗^	−0.925⁣^∗∗^	−0.969-⁣^∗∗^	−0.964-⁣^∗∗^
AchE	0.955⁣^∗∗^	0.921⁣^∗∗^	0.958⁣^∗∗^	1	−0.963⁣^∗∗^	−0.947⁣^∗∗^	0.963⁣^∗∗^	−0.937⁣^∗∗^	−0.972^−^⁣^∗∗^	−0.971-⁣^∗∗^
Folic acid	−0.964⁣^∗∗^	−0.925⁣^∗∗^	−0.959⁣^∗∗^	−0.963⁣^∗∗^	1	0.953⁣^∗∗^	−0.952⁣^∗∗^	0.953⁣^∗∗^	0.968⁣^∗∗^	0.978⁣^∗∗^
Vitamin B12	−0.923⁣^∗∗^	−0.927⁣^∗∗^	−0.941⁣^∗∗^	−0.947⁣^∗∗^	0.953⁣^∗∗^	1	−0.896⁣^∗∗^	0.955⁣^∗∗^	0.977⁣^∗∗^	0.954⁣^∗∗^
MDA	0.951⁣^∗∗^	0.917⁣^∗∗^	0.949⁣^∗∗^	0.963⁣^∗∗^	−0.952⁣^∗∗^	−0.896⁣^∗∗^	1	−0.907⁣^∗∗^	−0.945-⁣^∗∗^	−0.960-⁣^∗∗^
TAC	−0.915⁣^∗∗^	−0.896⁣^∗∗^	−0.925⁣^∗∗^	−0.937⁣^∗∗^	0.953⁣^∗∗^	0.955⁣^∗∗^	−0.907⁣^∗∗^	1	0.964⁣^∗∗^	0.954⁣^∗∗^
SOD	−0.946-⁣^∗∗^	−0.934-⁣^∗∗^	−0.969-⁣^∗∗^	−0.972-⁣^∗∗^	0.968⁣^∗∗^	0.977⁣^∗∗^	−0.945-⁣^∗∗^	0.964⁣^∗∗^	1	0.978⁣^∗∗^
CAT	−0.961-⁣^∗∗^	−0.932-⁣^∗∗^	−0.964-⁣^∗∗^	−0.971-⁣^∗∗^	0.978⁣^∗∗^	0.954⁣^∗∗^	−0.960-⁣^∗∗^	0.954⁣^∗∗^	0.978⁣^∗∗^	1

⁣^∗∗^Correlation is significant at the *p* < 0.05 level and *p* < 0.01 level.

**Table 2:** Therapeutic effects of the methanolic extract of *P. oleracea* on the transit time spent in the T-maze by different rat groups.

Groups	Biochemical parameters		
	First trial (sec.) baseline	Second trial (sec.) induction	Third trial (sec.) induction
GI (negative control group)	13.83 ± 0.70	13.43 ± 0.50^#^	12.75 ± 0.60^#^
GII (AD-positive control group)	13.7 ± 0.30	24.65 ± 1.92⁣^∗^	27.15 ± 0.76⁣^∗^
GIII (treated with 100 mg/kg of PO extract)	13.86 ± 0.47	20.60 ± 0.92⁣^∗#^	22.51 ± 0.91⁣^∗#^
GIV (treated with 200 mg/kg of PO extract)	14.00 ± 0.23	15.92 ± 0.45⁣^∗#^	18.64 ± 0.88⁣^∗#^

*Note:* The results were expressed as the M ± SD. For explaining the ∗ and the # (*p* < 0.05).

⁣^∗^Significant, *p* < 0.05 as compared to the control group.

^#^Significant, *p* < 0.05 as compared to the AD group.

## Data Availability

All the data are available on a kind request from the corresponding author.

## Nomenclature

ACh: Acetylcholine
AD: Alzheimer's disease
Aβ: Amyloid beta
CAT: Catalase
GI: Untreated negative control group
GII: The positive AD control group given aluminum chloride (AlCl_3_) (17 mg/kg body weight) daily for 45 days, until AD was induced
GIII: Rats were given AlCl_3_ as in GII and concurrently treated with *P. oleracea* methanolic extract at a dose of 100 mg/kg per day for 15 days
GIV: Rats were given AlCl_3_ as in GII and concurrently treated with 200 mg/kg of *P. oleracea* methanolic extract daily for 15 days
IL-6: Interleukin-6
MDA: Malondialdehyde
PO: *Portulaca oleracea*
ROS: Reactive oxygen species
SOD: Superoxide dismutase
TAC: Total antioxidant capacity
TNF-α: Tumor necrosis factor

## Supporting Information

Table S1: Serum concentrations of IL-6, TNF-α, Aβ, and AchE.

Table S2: Concentrations of oxidative stress parameters and antioxidant biomarkers.
