# Supplementary material for: Portulaca oleracea L. Methanolic Extract Protects the Brain of Male Rats Against Alzheimer's Disease
Source: Scientifica (Cairo). 2025 Feb 22;2025:7701263. doi: 10.1155/sci5/7701263 (PMC11871972; doi:10.1155/sci5/7701263)
Supplement: Supporting Information — Additional supporting information can be found online in the Supporting Information section. [file 7701263.f1.docx]

**Table S1.** serum concentrations of IL-6, TNF- α, Aβ and AchE.

| Groups  Biochemical parameters | IL-6  (pg/ml) | TNF-α  (pg/ml) | Aβ  (pg/ml) | AchE  (pg/ml) | Folic Acid  (pg/ml) | Vitamin B12  (pg/ml) |
| --- | --- | --- | --- | --- | --- | --- |
| GI  (negative Control group) | 11.26± 1.56# | 14.5± 2.48# | 43.25±  5.72# | 97.73± 6.84# | 86.85±  5.24# | 264.93±  2.77# |
| GII (AD positive control group) | 77.33±  2.79^*#^ | 57.7± 5.61^*^ | 105.61± 6.28^*^ | 176.11± 6.24 * | 34.88± 2.91^*^ | 128.98±  6.37 |
| GIII (treated with 100mg/kg of PO extract) | 55.15± 6.0^*^ | 39.0± 7.62^*#^ | 82.7±  3.28^*#^ | 152.03± 6.05*# | 49.78± 3.09^*^# | 170.66± 7.17^*^# |
| GIV  (treated with 200mg/kg of PO extract) | 20.11±  2.04^*#^ | 26.11±  2.80^*#^ | 59.78± 4.14^*#^ | 120.90± 4.58*# | 69.85± 3.89^*^# | 194.01± 4.55^*^# |

The results were expressed as the M ± SD. Showed a statistically significant difference (P < 0.05).

(*) Significant, p<0.05; as compared control group. (^#^) Significant, p< 0.05 as compared to AD group

**Table S2.** Concentrations of oxidative stress parameters and antioxidants biomarkers.

| Groups  Biochemical parameters | **TAC**  **(mM/L)** | **SOD**  **(U/L)** | **CAT**  **(U/L)** | **MDA**  **(nmol/ml)** |
| --- | --- | --- | --- | --- |
| GI  (negative Control group) | 3.51±0.21^#^ | 86.66 ± 3.54^#^ | 4.75±0.34^#^ | 0.46±0.07^#^ |
| GII (AD positive control group) | 0.37±.0.05^*^ | 17.55 ± 2.51^*^ | 0.53±0.03^*^ | 3.58±0.38^*^ |
| GIII (treated with 100mg/kg of PO extract) | 0.94±0.48^*#^ | 34.26 ± 3.40^*#^ | 1.70±0.09^*#^ | 2.48±0.29^*#^ |
| GIV  (treated with 200mg/kg of PO extract) | 2.05±0.20^*#^ | 58.66 ± 2.02^*#^ | 3.43±0.24^*#^ | 0.97±0.20^*#^ |

The results were expressed as the M ± SD. Showed a statistically significant difference (P < 0.05)

(*) Significant, p<0.05; as compared to the control group. (^#^) Significant, p< 0.05 as compared to the AD group.
